# Determinants of breastfeeding initiation and cessation among employed mothers: a prospective cohort study

**DOI:** 10.1186/s12884-016-0965-1

**Published:** 2016-07-29

**Authors:** Rada K. Dagher, Patricia M. McGovern, Jesse D. Schold, Xian J. Randall

**Affiliations:** 1 Department of Health Services Administration, School of Public Health, University of Maryland, College Park, MD USA; 2 Division of Environmental Health Sciences, University of Minnesota, Minneapolis, MN USA; 3 Department of Quantitative Health Sciences, Cleveland Clinic, Cleveland, OH USA; 4 US Department of Housing and Urban Development, Washington, DC USA

**Keywords:** Breastfeeding, Family leave policy, Postpartum, Workplace barriers

## Abstract

**Background:**

The U.S. continues to have one of the lowest breastfeeding rates in the industrialized world. Studies have shown that full-time employment and early return to work decreased breastfeeding duration, but little is known about the relationship between leave policies and breastfeeding initiation and cessation. This study aimed to identify workplace-related barriers and facilitators associated with breastfeeding initiation and cessation in the first 6 months postpartum.

**Methods:**

A prospective cohort study design was utilized to recruit 817 Minnesota women aged 18 and older while hospitalized for childbirth. Selection criteria included English-speaking, employed mothers with a healthy, singleton birth. These women were followed up using telephone interviews at 6 weeks, 12 weeks, and 6 months after childbirth. The main study outcomes were breastfeeding initiation, measured during hospital enrollment, and breastfeeding cessation by 6 months postpartum.

**Results:**

Women were 30 years old; 86 % were White, and 73 % were married. Breastfeeding rates were 81 % at childbirth, 67 % at 6 weeks, 49 % at 12 weeks, and 33 % at 6 months postpartum. Logistic regression revealed the odds of breastfeeding initiation were higher for women who: held professional jobs, were primiparae, had graduate degree, did not smoke prenatally, had no breastfeeding problems, and had family or friends who breastfeed. Survival analyses showed the hazard for breastfeeding cessation by 6 months was: higher for women who returned to work at any time during the 6 months postpartum versus those who did not return, lower for professional workers, higher among single than married women, higher for every educational category compared to graduate school, and higher for those with no family or friends who breastfeed.

**Conclusions:**

While employer paid leave policy did not affect breastfeeding initiation or cessation, women who took shorter leaves were more likely to stop breastfeeding in the first 6 months postpartum. Future research should examine women’s awareness of employer policies regarding paid and unpaid leave.

## Background

The U.S. continues to have one of the lowest breastfeeding rates in the industrialized world [1, 2]. Data from 2010 indicate that 76.5 % of U.S. mothers initiated breastfeeding, and only 49 % reported feeding any human milk to their infants at six months [3]. These figures fall short of the Healthy People (HP) 2020 goals of 81.9 % for initiation of breastfeeding and 60.6 % for any breastfeeding at 6 months [4]. Breastfeeding has established benefits for both maternal and child health [2]; however, employers may also benefit from supporting breastfeeding in the workplace. Employed mothers who breastfeed incur lower healthcare costs for themselves and their babies [5], and have lower workplace absenteeism than non-breastfeeding mothers [6]. Yet, often mothers find it difficult to continue breastfeeding after returning to work. About 70 % of employed mothers of infants younger than three years have full-time jobs and around one third of these mothers return to their jobs within the first three months postpartum [7]. Therefore, it is important to understand workplace-related barriers and facilitators to the initiation and continuation of breastfeeding.

The literature on employment characteristics has shown factors such as having a non-professional occupation [8, 9] and planning to work full-time after childbirth [10, 11] decreased the likelihood of breastfeeding initiation. Moreover, factors such as being employed full-time [11, 12] and early return to work after childbirth [13, 14] increased the likelihood of early breastfeeding cessation after childbirth, whereas having a professional job [9, 15], a flexible job [13, 14], and access to employer family-friendly benefits [13] decreased the likelihood of early breastfeeding cessation. However, scarce research has examined the impact of employer paid leave policies on breastfeeding.

The U.S. is the only industrialized country in the world that does not have a national paid maternity leave policy [16]. Having access to paid leave has been associated with longer leaves among mothers after childbirth [17]. The main federal law governing leave in the U.S. is the Family and Medical Leave Act (FMLA) of 1993, which mandates unpaid leave of 12 weeks for perinatal care, childbirth, and caring for a newborn or an adopted child [18]. Thus, it is not surprising that U.S. national data on first time mothers show that 58.6 % returned to paid work in the first 3 months postpartum [19]. Economic theory on the household production function [20], predicts that women may choose the duration of leave from work after childbirth as an input to the production of their child’s health (in this case through taking time off to breastfeed) subject to constraints such as relatively low household income and employer paid/unpaid leave policies.

The objectives of this study are to identify the rates of breastfeeding among employed women during the first 6 months after childbirth and to investigate the association of employer paid leave policies and other employment characteristics with the initiation and cessation of breastfeeding by 6 months postpartum. This study contributes to the literature by focusing exclusively on employed mothers in the state of Minnesota, which has one of the highest female labor force participation rates in the nation (71.2 %) but does not have a statewide paid leave policy [21], and identifying the influence of employer paid leave policies on breastfeeding initiation and cessation.

## Methods

The Maternal Postpartum Health Study is a prospective cohort study that recruited employed Minnesota women while hospitalized for childbirth [22]. The study population included women ages 18 and older and admitted to one of 3 community hospitals in Minneapolis and St. Paul for childbirth in 2001. Recruitment occurred between April 9, 2001 and November 19, 2001. According to vital statistics data, recruited mothers were comparable on demographics and birth characteristics to mothers who delivered at 41 other hospitals in the seven-county metropolitan area [22]. Sample selection criteria included: having had a healthy singleton infant, English-speaking, prenatally employed for at least 20 h per week, planning to continue employment after childbirth, and planning to keep the infant.

**Fig. 1:**
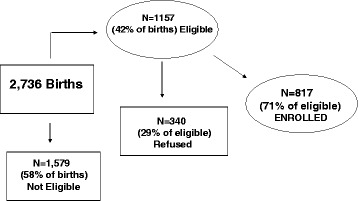
Flow diagram depicting participation rate and eligibility for the Maternal Postpartum Health Study

Among 2,736 women giving birth at the 3 study hospitals for the enrollment period, 1157 met our sample selection criteria (42 % of all births). Of these 1157 women, 817 agreed to participate in the study and were enrolled in the hospital (response rate: 71 %) (see Fig. 1). The primary reasons for nonparticipation were concerns about time commitment and lack of interest. Study participants were compared to women who refused participation to identify systematic bias using limited data from hospital medical records and interviews. Using a *t*-test of differences in the means, there were no significant differences between participants and refusals in regards to infant birth weight, gestational age, maternal age, marital status and years of employment. In addition, subjects were given gift certificates of $5 for completion of questionnaires at each data collection period to enhance subject compliance.

Telephone interviews of 45 min each were conducted at 6 weeks, 12 weeks, and 6 months after delivery. The follow up telephone interviews used a four week window for conducting interviews at each of 6 weeks (i.e., 4 to 8 weeks) and 12 weeks (10 to 14 weeks) postpartum and an 8 week window at 6 months (5 to 7 months) postpartum. Out of the 817 recruited women, 716 (88 %) completed the 6 weeks interview, 661 (81 %) completed the 12 weeks interview, and 625 (76 %) completed the 6 months interview. Extensive training in non-biased interviewing techniques was provided to all University of Minnesota research staff who conducted the interviews. All interviews from 6 weeks to 6 months were completed by computer-assisted telephone interviewing (CATI) which does not allow the interviewer to skip a question.

Additionally, information was gathered from a random subsample of 195 women (25 % of the participants) to request their permission for contacting human resources representatives for purposes of validation of self-report information concerning the employer’s written family and medical leave policies. Trained staff conducted a 15 min telephone interview with employers to collect information on characteristics of written leave policies, including: paid vacation and sick leave, paid maternity leave, and any unpaid parental, family or medical leave.

### Study measures

Dependent variables: Breastfeeding initiation was asked during hospital enrollment [23], “Are you feeding your newborn: a) Breast milk only, b) Infant formula only, c) Some combination of breast milk and infant formula.” Any breastfeeding was coded as 1 in logistic regression analyses by combining responses a and c. Breastfeeding at 6 weeks, 12 weeks, and 6 months after childbirth was asked during telephone interviews, “Are you currently feeding your baby: a) breast milk, b) formula, c) milk (cow or soy), other?”. Among women who initiated breastfeeding, any breastfeeding was coded as 1 and considered as a time-to-event response in the survival analysis. Models were censored at six months following breastfeeding initiation. The abovementioned questions assessing breastfeeding used the definitions of breastfeeding that were in clinical use in the recruitment hospitals at the time. The independent variables included measures of employment characteristics, and the control variables (confounding factors) included measures of personal and perinatal factors as described in Table 1 [22–30].

**Table 1:** Measures of independent variables^*+^

Independent variables (Coding)	Item description, reference and data source
PERSONAL FACTORS^**^	
Age (years)	Abstracted from the medical chart by maternity nurses and calculated from date of birth. (Continuous Variable; Range: 18–45)
Race (1 = non-white, 0 = white)	Adapted from Census 2000 [24]^*^
Educational Status (High School Education or less = 1; else = 0; 2-year College/Technical = 1; else = 0; College Graduate = 1; else = 0; Graduate School = reference)	Adapted from the National Health Interview Survey [25]^*^
Marital Status (Single = 1; else = 0; Partnered = 1; else = 0; Married = reference)	Adapted from National Health Interview Survey [25]^+^
Parity (1 = Primipara; 0 = else)	Adapted from National Health Interview Survey [25]^*^
Annual Household Income ($)	Adapted from National Health Interview Survey [25]^*^
PERINATAL FACTORS^**^	
Prenatal Smoking (1 = no; 0 = yes)	“Did you smoke cigarettes during this pregnancy?”, item adapted from Palermo; [26]^*^
Pre-pregnancy Health (1 = poor/fair, 2 = good, 3 = very good, 4 = excellent)	“How would you rate your health in general before this pregnancy?”^*^
Prenatal Moods (1 = no; 0 = yes)	“During this pregnancy did you ever have a problem with your mood, such as feeling depressed or anxious?”, item taken from McGovern et al.; [23]^*^
Breastfeeding by Family and Friends (1 = no; 0 = yes)	“To the best of your knowledge, did any of your family or close friends breastfeed?” ^+^
Breastfeeding Problems (1 = yes; 0 = no)	“Have either you or your baby had any problems or conditions that may prevent you from breastfeeding?” ^*^ Women who answered “yes” received a follow up question: “What is the nature of the problem or condition?”^*^
Delivery Hospital (North Memorial = 1; else = 0; St. Joseph = 1; else = 0; St. John = reference)	
EMPLOYMENT CHARACTERISTICS	
Occupational Classification (Blue Collar/Service = 1; else = 0; Professional = 1; else = 0; Clerical = reference)	Taken from US Census [27]^+^
Leave Status (1 = working, 0 = on leave from work). Time-dependent covariate in survival models. Subjects were coded as 0 until the day they returned to work at which point they were coded as 1.	“Are you: 1. On leave (including part-time leave)? 2. Working again (whether from home or at the office)?” Item adapted from Cantor et al. [28] and asked at each of the three postpartum periods.
Employer Provides Paid Leave Policy (1 = yes, 0 = no)	“Are you eligible for any PAID time away from work with this employer (e.g., vacation or sick time, PTO or maternity/disability leave)?”^*^
Longest Paid Leave Possible by Empl oyer Policy (days)	“Assume you hadn’t used any sick leave or vacation this year. What is the longest leave you could have taken before and after childbirth and still received at least some pay?”^+^ (Continuous Variable; Range: 0–273)
Prenatal Hours Worked/Week (hrs)	Average work hours in the past 12 months^*^ (Continuous Variable; Range: 20–80)
Prenatal Job Stress (two item summary score of 2 = almost never to 10 almost always)	Items taken from Mardburg et al.; [29] “How often do you have too much to do? How often do you experience stress from your job?” ^*^ (Continuous Variable; Range: 0–8)
Supervisor Support (1 = Somewhat/Strongly disagree, 0 = Somewhat/Strongly agree)	Adapted from Bond et al.; [30] item asked: “My supervisor has been helpful to me when I have had to take care of personal or family matters.”^*+^
Coworker Support (1 = Somewhat/Strongly disagree, 0 = Somewhat/Strongly agree)	Adapted from Bond et al.; [30] item asked: “My coworkers have been supportive of me when I had to take care of personal or family matters.”^*+^

*Note*. This table is adapted from table 1 in an earlier publication [22]

^*^The asterisk denotes self report data collected in-person at enrollment in the hospital

^+^The cross denotes self report data collected by telephone at the six-week interview

^**^Personal and Perinatal factors were considered confounders (control variables) in logistic and survival analyses

### Data analyses

Our analytical sample included all 817 women. All analyses were conducted with SAS, v9.4 (Cary, N.C.).We first conducted descriptive statistics using means and frequencies (Table 2). Since the interviews were done using CATI, there were very few questions with missing data (7 % of 817 responses for income and < 2 % for the remaining variables). The only variable that needed to be addressed with imputation due to missing data was income. The study participants were asked a question about household income in two ways—(1) actual total household income in the year prior to childbirth (continuous), and (2) by broad income categories (discrete). About 10 % of women chose to answer by categories which we then imputed at the mean of the category. About 5 % of the women refused the income questions so we used regression imputation. A regression model was estimated using women similar by education, marital status and race to predict observed values of income for women’s missing information. Fitted values from the regression model were then used to impute the missing values.

**Table 2:** Sample characteristics (*N* = 817)

Variable	Frequency (%)	Mean (SD)
Maternal age (Years)		29.63(5.42)
Race		
White	681(83.4)	
Non-white	136(16.6)	
Educational Status		
High School or less	196(24.0)	
2-year College/Technical	267(32.7)	
College Graduate	267(32.7)	
Graduate School	87(10.6)	
Marital Status		
Single	75(10.5)	
Partnered	117(16.3)	
Married	524(73.2)	
Parity		
Primipara	370(45.3)	
Multipara	447(54.7)	
Annual Household Income ($)		70,538.14(38,010.80)
Prenatal Smoking		
Yes	123(15.1)	
No	694(84.9)	
Pre-pregnancy Health		
Poor/fair	20(2.4)	
Good	166(20.3)	
Very good	356(43.6)	
Excellent	275(33.7)	
Prenatal Moods		
Yes	385(47.1)	
No	432(52.9)	
Breastfeeding by Family and Friends		
Yes	607(84.8)	
No	109(15.2)	
Breastfeeding Problems		
Yes	48(5.9)	
No	769(94.1)	
Delivery Hospital		
North Memorial	340(41.6)	
St. Joseph	122(14.9)	
St. John	355(43.5)	
Occupational Classification		
Blue Collar/Service	103(14.4)	
Professional	332(46.4)	
Clerical	281(39.2)	
Employer Provides Paid Leave Policy		
Yes	615(76.1)	
No	193(23.9)	
Longest Paid Leave Possible by Employer Policy (days)		46.41(39.72)
Prenatal Hours Worked/Week (hrs)		38.13(8.39)
Prenatal Job Stress (summary score)		4.31(1.76)
Supervisor Support		
Somewhat/Strongly agree	644(89.9)	
Somewhat/Strongly disagree	72(10.1)	
Coworker Support		
Somewhat/Strongly agree	703(98.2)	
Somewhat/Strongly disagree	13(1.8)	

Next, we ran t-tests and chi-square tests to compare the characteristics of women who initiated breastfeeding to those who did not (Table 3). These constitute unadjusted analyses. Following that, we conducted a logistic regression analysis that adjusts for potential confounders, to examine the employment factors associated with breastfeeding initiation (Table 4). We tested and ascertained the assumption of no multicollinearity for logistic regression and assessed model fit.

**Table 3:** Bivariate baseline comparisons between women who initiated breast feeding and those who did not (*N* = 716)

Demographics and personal characteristics	Initiated breastfeeding (*N* = 580)	Did not initiate breastfeeding (*N* = 136)	*P*-value
Age at delivery, y			
Mean (SD)	30.15(5.28)	28.82(5.16)	0.008
Marital Status			
*N* (%)			<0.001
Married	443(84.5)	81(15.5)	
Partnered	94(80.3)	23(19.7)	
Single	43(57.3)	32(42.7)	
Educational Status			
*N* (%)			<0.001
High School or Less	104(66.2)	53(33.8)	
2-year College/Technical	182(79.8)	46(20.2)	
College Graduate	214(86.3)	34(13.7)	
Graduate School	80(96.4)	3(3.6)	
Race			
*N* (%)			<0.001
White	510(82.9)	105(17.1)	
Non-White	70(69.3)	31(30.7)	
Household Income ($)			
Mean (SD)	74,510.35(38,687.18)	59,934.49(32,599.82)	<0.001
Occupation			
*N* (%)			<0.001
Blue Collar	70(69.3)	31(30.7)	
Clerical	209(75.5)	68(24.5)	
Professional	297(89.5)	35(10.5)	
Total # hours worked			0.454
Mean (SD)	37.97(8.68)	38.58(7.96)	
Employer Provides Paid Leave Policy			
*N* (%)			0.040
No	119(75.3)	39(24.7)	
Yes	455(82.6)	96(17.4)	
Supervisor Support			
*N* (%)			0.462
Somewhat/Strongly Agree	524(81.4)	120(18.6)	
Somewhat/Strongly Disagree	56(77.8)	16(22.2)	
Coworker Support			
*N* (%)			0.738
Somewhat/Strongly Agree	569(80.9)	134(19.1)	
Somewhat/Strongly Disagree	11(84.6)	2(15.4)	
Parity Status			
*N* (%)			0.002
Multiparous	294(76.8)	89(23.2)	
Primiparous	286(85.9)	47(14.1)	
Had Prenatal Moods			
*N* (%)			0.886
Yes	269(80.8)	64(19.2)	
No	311(81.2)	72(18.8)	
Smoked Prenatally			
*N* (%)			<0.001
Yes	58(59.8)	39(40.2)	
No	522(84.3)	97(15.7)	
Delivery Hospital			
*N* (%)			0.974
St. John	262(81.4)	60(18.6)	
North Memorial	231(80.8)	55(19.2)	
St. Joseph	87(80.6)	21(19.4)	
Breastfeeding by Family and Friends			
*N* (%)			<0.001
Yes	519(85.5)	88(14.5)	
No	61(56.0)	48(44.0)	
Had Breastfeeding Problems			
*N* (%)			<0.001
Yes	19(50.0)	19(50.0)	
No	561(82.7)	117(17.3)	
Pre-pregnancy health			
*N* (%)			0.469
Fair/Poor	13(72.2)	5(27.8)	
Good	112(78.9)	30(21.1)	
Very Good	250(80.4)	61(19.6)	
Excellent	205(83.7)	40(16.3)	

*Note. P*-values are presented for chi-square statistics and t-tests; alpha <0.05 was used

**Table 4:** Results of the logistic regression predicting breastfeeding initiation^b^

Employment variables	β	SE^a^	*P*-value	OR	CI
Type of Occupation (Ref. = Clerical)					
Professional	0.53	0.28	0.05	1.70	1.00–2.93
Blue Collar	−0.23	0.30	0.44	0.79	0.44–1.43
Prenatal Hours Worked per Week	−0.02	0.01	0.21	0.98	0.96–1.01
Employer Provides Paid Leave Policy (Ref. = No)	−0.29	0.28	0.31	0.75	0.43–1.31
Supervisor Support (Ref. =Somewhat/Strongly agree)	0.02	0.35	0.96	1.02	0.51–2.03
Coworker Support (Ref. = Somewhat/Strongly agree)	−0.02	0.88	0.98	0.98	0.17–5.49

^a^Standard Error

^b^Model was adjusted for maternal age, race/ethnicity, marital status, education, household income, delivery hospital, parity, prenatal smoking, prenatal moods, breastfeeding among friends/family, had problems with breastfeeding, and pre-pregnancy health; alpha <0.05 was used

Finally, Cox proportional hazards analysis was used to model the likelihood of breastfeeding cessation during the 6 months after childbirth as a function of employment circumstances, adjusting for potential confounders (Table 5). The Cox models were right censored at the time of study participant’s last follow up. The results were presented as coefficients and hazard ratios (HR). To test the assumption of proportional hazards in the Cox model, we utilized the global test [31] as well as visual inspection of the plot of Schoenfeld residuals as a function of time to detect non-zero slopes [32]. We did not find evidence of any statistically significant departures for the assumptions of the Cox models. We used alpha <0.05 for all the statistical analyses in this paper.

**Table 5:** Results of the cox proportional hazards regression predicting breastfeeding cessation^c^

	Analysis of maximum likelihood estimates				
Parameter	Parameter Estimate	SE^a^	Chi-Square	*P* Value	Hazard Ratio (95 % CI)
Supervisor Support (Ref. = Somewhat/Strongly Agree)	0.02	0.15	0.02	0.88	1.02 (0.76–1.38)
Coworker Support (Ref. = Somewhat/Strongly Agree)	0.26	0.34	0.59	0.44	1.30 (0.67–2.52)
Prenatal Job Stress	0.01	0.03	0.05	0.83	1.01 (0.95–1.06)
Prenatal Hours Worked per Week	−0.04e^−3^	0.01	0.00	0.99	1.00 (0.99–1.01)
Leave Status (Ref = On Leave from Work)^b^	0.38	0.13	9.16	<0.01	1.46 (1.14–1.87)
Longest Paid Leave Possible (days)	0.11e^−2^	0.12e^−2^	0.76	0.38	1.00 (0.99–1.00)
Type of Work (Ref. = Clerical)					
Professional	−0.34	0.12	8.78	<0.01	0.71 (0.56–0.89)
Blue Collar	−0.03	0.13	0.06	0.81	0.97 (0.75–1.26)

^a^Standard Error

^b^Time-dependent covariate

^c^Model was adjusted for maternal age, race/ethnicity, marital status, education, household income, delivery hospital, parity, prenatal smoking, prenatal moods, breastfeeding among friends/family, and pre-pregnancy health; alpha <0.05 was used

The confounding variables chosen for the logistic regression and survival analyses were selected based on an a priori causal theoretical model and directed acyclic graphs that take into account the factors associated with initiation of breastfeeding and cessation of breastfeeding. This method is described by Greenland et al. [33] and illustrated by Hernan et al. [34]. These confounding factors include the personal (maternal age, race/ethnicity, marital status, education, household income, delivery hospital, and parity) and perinatal characteristics (prenatal smoking, prenatal moods, breastfeeding among friends/family, had problems with breastfeeding, and pre-pregnancy health) presented in Table 1.

## Results

### Descriptive characteristics

From the sample of *N* = 817, on average, women were 30 years old, 83 % were White, 73 % were married and 16 % lived with their partner, 33 % had two years of college or technical school, 33 % were college graduates and 11 % had a graduate degree (Table 2). Moreover, 45 % were primiparous, 15 % smoked prenatally, 47 % experienced prenatal moods of depression or anxiety, and 77 % reported excellent/very good health before pregnancy. Most women had family or friends who breastfeed (85 %), and only a minority experienced problems that may prevent them from breastfeeding (5 %). Problems mothers experienced with breastfeeding included having had breast reduction surgery (*n* = 5), a size or shape of the breast or nipple that was a barrier for infant latching or sucking (*n* = 5), taking medications or individual health issues (*n* = 13), and not producing enough milk (*n* = 2). Problems infants experienced with breastfeeding included premature or preterm birth (*n* = 4), poor latching or sucking (*n* = 5), medical condition that impeded nursing (*n* = 4), and a sleepy baby (*n* = 2). Rates of any breastfeeding were 81 % at childbirth, 67 % at 6 weeks, 49 % at 12 weeks, and 33 % at 6 months postpartum.

In terms of employment characteristics, women worked on average 38 h per week during pregnancy, 46 % worked in professional occupations and 39 % in clerical occupations, 76 % worked for employers who provide some type of paid leave, and 96 % planned to return to work after childbirth (4 % were unsure). The percentage of women who were back to work was 7 % at 6 weeks postpartum, 47 % at 12 weeks postpartum, and 86 % at 6 weeks postpartum. Moreover, 90 % somewhat or strongly agreed that their supervisor was supportive during their pregnancy and when arranging for maternity leave and 98 % somewhat or strongly agreed that their co-workers were supportive.

### Bivariate analyses results

Table 3 compared women who initiated breastfeeding with non-initiators by demographic and personal characteristics. Breastfeeding initiators were significantly more likely to have a professional occupation and to work for an employer that provides paid leave than women who did not initiate breastfeeding. They were also more likely to be white, older, married or living with partner, primiparous, have college degree or higher, have a higher household income, did not smoke during pregnancy, have family or friends who breastfed, and had no breastfeeding problems as compared with non-initiators of breastfeeding.

### Logistic regression results

Logistic regression (Table 4), adjusted for confounders, revealed that the odds of breastfeeding initiation were higher for professional than for clerical workers [Odds Ratio (OR) =1.70; Confidence Interval (CI): 1.00, 2.93]. However, there were no significant associations between breastfeeding initiation and other employment variables including number of hours worked during pregnancy, whether the employer provides paid leave, prenatal supervisor support, and prenatal coworker support.

Other significant findings in the logistic regression showed that the odds of breastfeeding initiation were significantly higher for women who were primiparae (OR = 1.82; CI: 1.13, 2.95), did not smoke prenatally (OR = 2.13; CI: 1.19, 3.83), and had no breastfeeding problems (OR = 4.88; CI: 2.28, 10.46), but were significantly lower for those with high school education or less (versus graduate school) (OR = 0.21; CI: 0.05, 0.80) and those who lacked family or friends who breastfed (OR = 0.30; CI: 0.18, 0.51).

### Survival analyses results

**Fig. 2:**
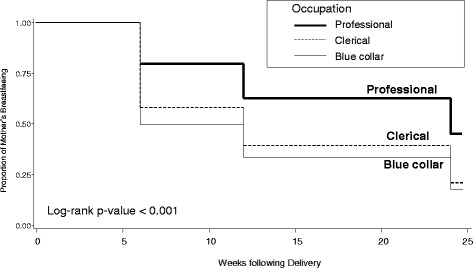
Kaplan-Meier plot of the rate of cessation of breastfeeding in the first 6 months by the woman’s occupational status

Results of the likelihood ratio test of the Cox model indicated a statistically significant association of covariates for the primary outcome (*p* < 0.001). Cox proportional hazards regression (Table 5), adjusted for confounders, showed that the hazard for breastfeeding cessation during the first 6 months after childbirth among women who initiated breastfeeding was higher for women who returned to work at any time during the 6 months postpartum [Hazard Ratio (HR) = 1.46; CI: 1.14, 1.87] compared to those who did not return, and was lower for professional (versus clerical) workers (HR = 0.71; CI: 0.56, 0.89). However, there were no significant associations between breastfeeding cessation and other employment factors including the number of hours worked prenatally, longest paid leave possible by employer policy, supervisor and coworker support at 6 weeks postpartum, and job stress score. Unadjusted Kaplan Meier plot of breastfeeding cessation during the first 6 months after childbirth by mother’s occupation was depicted in Fig. 2. It showed a higher rate of breastfeeding cessation among women who were in blue collar occupations, followed by those in clerical occupations, and then professional occupations (*P* <0.001).

Other significant findings in the Cox proportional hazards regression showed that the hazard of breastfeeding cessation were: 1) higher among single (HR = 1.39; CI: 1.01, 1.91) than married women, 2) higher among those with high school education or less (HR = 1.97; CI: 1.26, 3.08) than those with graduate school, higher among those with junior college (HR = 1.61; CI: 1.06, 2.44) than those with graduate school and higher among those with a college degree (HR = 1.50; CI: 1.03, 2.20) than those with graduate school, and 3) higher for those who lacked family or friends who breastfed (HR = 1.61; CI: 1.27, 2.04).

## Discussion

The sample of employed mothers in this study had a breastfeeding initiation rate of 81 %, which exceeded HP 2010 goals of ever breastfeeding (75 %) [35], and came very close to HP 2020 goals of ever breastfeeding (81.9 %) [4]. In our sample, the rate of any breastfeeding at 6 months was 33 %, which fell below HP goals for any breastfeeding at 6 months (HP 2010 goals: 50 %; HP 2020 goals: 60.6 %) [4, 35]. These results are consistent with the literature suggesting the challenges for employed women to breastfeed for 6 month duration [36], and the potential need for interventions to support employed women.

This is one of the first studies to examine whether access to paid leave through employer policy is associated with improved breastfeeding. Having an employer that provides paid leave was not associated with breastfeeding initiation and the longest paid leave available to a woman given her employer’s policy benefits was not associated with breastfeeding cessation during the 6 months after childbirth. In a sub study where we interviewed 90 employers representing 118 women in our sample about their leave policies, we found that only 29 % to 35 % of women’s self-report data matched their employers’ reports of total duration of available job-protected leave, paid and unpaid [36]. These findings are consistent with national findings on employees’ general awareness of their leave eligibility under the FMLA [28]. Thus, employers that solely institute paid leave policies without ensuring their employees’ awareness of these policies may not improve breastfeeding rates among new mothers. This may potentially explain why in our study the duration of paid leave per employer policy was not associated with breastfeeding duration while duration of leave taken by the mother was associated with breastfeeding duration. Future research should explore potential strategies employers can use to improve their employees’ awareness of their leave policies. It is also possible that women have access to some paid leave but do not use it because of stigma or only partial subsidized leave.

Conversely, the hazard for breastfeeding cessation during the first 6 months after childbirth was higher for women who returned to work at any time during the 6 months postpartum compared to those who did not return. The positive association between maternity leave duration and breastfeeding duration is consistent with other studies [13–15]. The association between return-to-work and early breastfeeding cessation highlights some role incompatibility between breastfeeding and employment. This suggests the importance of having employer support for breastfeeding practices by encouraging women to utilize their paid leaves, instituting lactation support programs in the workplace, and following the provisions of Section 4207 of the Affordable Care Act (ACA) in accommodating breastfeeding mothers in the workplace [37]. As part of the ACA of 2010, employers are required to provide employees with a reasonable break time to express breast milk for her nursing child for one year after the child’s birth; and a place for expressing breast milk, that is shielded from view and free from intrusion from coworkers and the public [37]. Primary care providers and occupational health nurses could work with pregnant and postpartum women to identify barriers and potential solutions to enhance breastfeeding outcomes in the workplace.

The findings that professional women had a higher likelihood of breastfeeding initiation and a lower likelihood of breastfeeding cessation compared to clerical women are consistent with other studies [9]. Women in professional occupations generally have greater job autonomy which affords them more privacy and more flexibility to accommodate the timing and place requirements of breastfeeding [38]. Professional women may also enjoy better access to employer-sponsored lactation programs than women in non-professional occupations, sometimes within the same workplace [39]. Thus, occupational health or human resources personnel who design worksite intervention programs that encourage breastfeeding upon return to work after childbirth would be well served to seek input from women across occupational categories to understand any potentially unique needs based upon job class, schedules and locations. Future research should investigate the mechanisms (e.g., job flexibility, access to private space, workplace support, or leave policies) through which having a professional occupation influences breastfeeding initiation and continuation.

Similar to prior research, we found that mothers who do not initiate breastfeeding are more likely to be multiparae [40], have a high school education or less [41], have smoked during pregnancy [42], have no friends or family who breastfeed [43], and to have experienced breastfeeding problems [44]. Also consistent with the literature, mothers who stopped breastfeeding during the first 6 months after childbirth were more likely to be single [41], less educated [45], and have no friends or family who breastfeed [43]. These findings are helpful in identifying specific target groups for public health interventions to promote breastfeeding initiation and continuation. For example, primary care providers could inform pregnant women on the effects of smoking on breastfeeding success and refer them to smoking cessation programs as early as feasible before childbirth. It is also important to provide women access to lactation counselors who can teach them strategies to prevent breastfeeding problems and effective management of these problems if they arise. In exploratory analyses, we found that women who had no family or friends who breastfeed had significantly lower income, were less likely to be college-educated and more likely to be in clerical and blue collar jobs than those who had family or friends who breastfeed (results available upon request). Thus, lack of family or friends who breastfeed can be a marker that identifies underprivileged groups of women and may help primary care providers recognize women who require more support and guidance for breastfeeding and in turn connect them with support groups for breastfeeding such as La Leche League International [46] and other breastfeeding peer support groups. Future research should explore whether breastfeeding by family and friends can be effective as a flag for obstetricians and nurses to recognize women who may need additional education and support in prenatal care, in the hospital at childbirth, and in postpartum care.

### Limitations

The study findings should be interpreted in light of its limitations. The results of this study can mainly be generalized to employed women of similar racial and ethnic backgrounds and income levels. Given that majority of the sample was white and of middle to high socioeconomic status, mirroring the socio-demographics of the Twin Cities area when the study was conducted, they may have had access to resources (such as maternity leave and childcare) that are not readily available for many new mothers in the U.S., which may in turn affect breastfeeding. This suggests the importance of replicating this study on a more diverse sample of mothers in other states. The study was conducted in 2001, yet the findings are still relevant 15 years later due to the fact that there have been no changes to the FMLA or to the Minnesota state leave law both of which continue to only stipulate unpaid leave. However, future studies should evaluate whether the breastfeeding provisions of the ACA have resulted in any changes in the reported relationships in this study. Around 96 % of the women planned to return to work after childbirth and 4 % were unsure, thus we could not examine the relationship between return-to-work plans and breastfeeding initiation. In addition, since the exact timing of breastfeeding cessation was not known, the analyses could only incorporate the events at discrete follow up time points (6 weeks, 12 weeks, and 6 months postpartum). This lack of more granular information would tend to dilute effects assuming the associations were more likely to occur early in the applicable intervals. As such, the results may under represent the degree of significant associations and similarly result in null findings for effects that may have been significant with more specific information. We only had information on leave status at key intervals (6 weeks, 12 weeks and 6 months) rather than a specific day of return to work. Due to this lack of precision, the effect of leave status may have been underrepresented as the actual day of return was generally sooner than measured in the questionnaire. The length of the survey instrument precluded inclusion of multi-item measures of coworker and supervisor support which would have been more reliable and stable measures of these constructs. Moreover, these variables had low variability with most women somewhat or strongly agreeing that they had coworker and supervisor support; thus statistical power was reduced. While maternity nurses were instructed to double check whether the mothers who answered yes to the breastfeeding initiation question were actually breastfeeding, a few mothers could have said they were breastfeeding but changed their minds and thus positive responses to this question may be over-reported. Since the question regarding breastfeeding initiation could be interpreted as intent to breastfeed without actual evidence of breastfeeding initiation, our results could be altered if there are systematic differences of characteristics of women that were likely to report intent but did not carry out breastfeeding. This potential bias should be considered with inferences from our analyses. Given the observational study design, causal relationships between employment characteristics and breastfeeding variables could not be ascertained and omitted variables such as intent to breastfeed or partner support which could confound the relationship between leave and breastfeeding could not be accounted for*.*

## Conclusion

Our findings show that having a professional occupation had positive effects on breastfeeding initiation and continuation. Moreover, not returning to work in the first 6 months after childbirth was associated with lower likelihood of breastfeeding cessation. Given that a substantial amount of employed women return to work in the first three months postpartum, work policies that support longer duration of breastfeeding in line with HP2020 goals are warranted. Additionally, research is needed to identify the structural components of a professional job that facilitate maternal breastfeeding and could potentially be extended to women employed in nonprofessional occupations. Intervention programs that encourage breastfeeding should specifically target at-risk populations of women, including the single, less educated, those in non-professional occupations, those who smoked during pregnancy, and women with no family or friends who breastfeed.

## Abbreviations

ACA, affordable care act; FMLA, family and medical leave act; HP, healthy people
